# Association between Dietary Patterns and Depression in Chinese Older Adults: A Longitudinal Study Based on CLHLS

**DOI:** 10.3390/nu14245230

**Published:** 2022-12-08

**Authors:** Zhongfei Pei, Jiajun Zhang, Wenzhe Qin, Fangfang Hu, Yan Zhao, Xiaohong Zhang, Xinxia Cong, Chuanli Liu, Lingzhong Xu

**Affiliations:** 1 Centre for Health Management and Policy Research, School of Public Health, Cheeloo College of Medicine, Shandong University, Jinan 250012, China; 2 National Health Commission Key Laboratory of Health Economics and Policy Research, Cheeloo College of Medicine, Shandong University, Jinan 250012, China; 3 Shandong University Center for Health Economics Experiment and Public Policy Research, Jinan 250012, China

**Keywords:** older adults, dietary pattern, depression, cohort study

## Abstract

(1) Objective: This study aimed to investigate the relationship between dietary patterns and depression in Chinese older adults. (2) Method: A cohort study was conducted on the relationship between dietary patterns and the risk of depression in older adults based on the China Health and Longevity Longitudinal Survey (CLHLS) from 2011 to 2014. Exploratory factor analysis was used to identify dietary patterns. The relationship between dietary patterns and the risk of depression after four years was examined using logistic regression, and subgroup analysis was carried out to determine whether the association differed by gender. (3) Results: A total of 2873 older adults were included in our cohort study. Three dietary patterns were identified: vegetable–egg–bean–milk pattern, meat–fish pattern, and salt-preserved vegetable–garlic pattern. The vegetable–egg–beans–milk pattern was negatively correlated with the risk of geriatric depression development (adjusted OR = 0.65 (95%CI: 0.49–0.87)), and the salt-preserved vegetable–garlic pattern was positively associated with aged depression risk (adjusted OR = 1.33 (95CI: 1.00–1.77)). The meat–fish pattern was not associated with the risk of depression in older adults. These associations were consistent in both men and women. (4) Conclusions: In this cohort study, the vegetable–egg–beans–milk dietary pattern was associated with lower risk of depression, while the salt-preserved vegetable–garlic dietary pattern was associated with higher risk of depression, and there were no gender differences in these associations.

## 1. Introduction

Population aging has become a key social issue in China. According to the seventh National Census statistics, China’s population aged 60 and above has reached 260 million, accounting for 18.7% of the total population, with 190 million aged 65 and above accounting for 13.5% of the total population [1]. The proportion of the elderly aged 65 and above in the total population is expected to rise to around 26.9% by 2050 [2]. The health of older adults needs urgent attention in the context of aging.

Depression refers to emotional disorders centered on low mood, mainly manifesting as sadness, emptiness or irritability, accompanied by various cognitive and physical symptoms, and it is one of the common mental disorders in the elderly [3]. In China, 4.46% of the elderly suffer from depression, while 35.19% have depressive symptoms [4]. Geriatric depression has a detrimental impact on the elderly’ quality of life and raises their care demands. It also promotes suicide behavior, resulting in primary unfavorable health outcomes, bringing a significant burden to families and society [5]. Therefore, the problem of depression in the elderly cannot be overlooked.

Compared with young people, the condition of depression in the elderly is more likely to be modified by lifestyle factors [6,7]. As a modifiable lifestyle, diet is an essential determinant of old-age health and well-being [8]. Since diet can affect some physiological mechanisms underlying depression, such as inflammation, oxidative stress, brain plasticity, function and stress-response systems [9], diet may play a significant role in the onset and progression of depression.

Many studies have shown the association of certain foods or nutrients with depression [10,11,12,13,14]. However, in real life, people usually eat a diet of multiple food combinations rather than isolated nutrients or foods because the components of dietary patterns may interact with each other and produce a synergistic effect, which may more effectively control depression [15]. Previous studies have explored the relationship between some dietary patterns and depression [16,17,18,19,20,21,22,23,24]. However, diet is culturally specific, and existing studies’ conclusions may not apply to the Chinese population. At the same time, there were limited data on the association between dietary patterns and depression in China. Most relevant studies focused on adults or were limited by cross-sectional designs [21,22,23,24].

Due to the limited studies, we explored the longitudinal association between dietary patterns and the risk of depression in Chinese older adults based on the Chinese Longitudinal Health and Longevity Survey (CLHLS) cohort data from 2011 to 2014.

## 2. Materials and Method

### 2.1. Data Source and Subjects

The data in this cohort study were derived from the Chinese Longitudinal Healthy Longevity Survey (CLHLS, 1998–2018). The CLHLS is an ongoing prospective cohort study of the elderly aged 60 years and above. A multi-stage cluster random sampling method was used to survey the elderly aged 60 and above and their adult children in 23 provinces of China. The baseline survey was conducted in 1998, followed by follow-up surveys in 2000, 2002, 2005, 2008–2009, 2011–2012, 2014 and 2017–2018.

**Figure 1:**
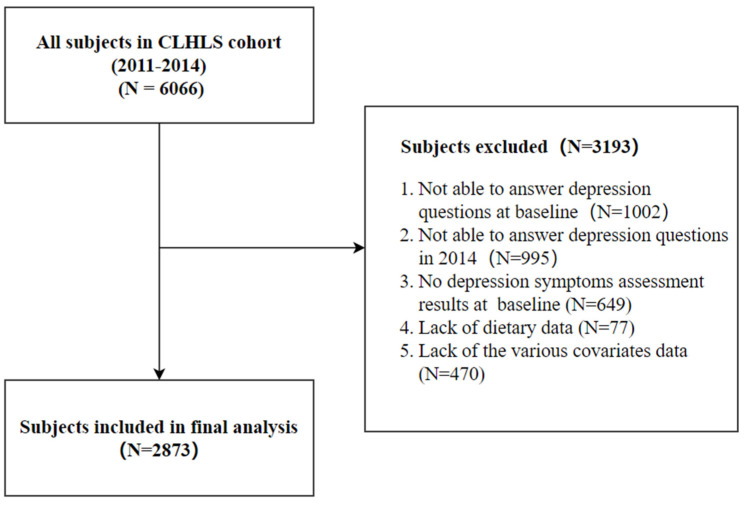
Selection process of subjects.

To reflect the latest dietary patterns and depression situation of Chinese older adults in CLHLS, our cohort study utilized the data from 2011 to 2014 due to the modification of the depression scale in the 2018 CLHLS questionnaire, which resulted in the inconsistency between the 2014 and 2018 depression scales. The study included dietary-related data, depression, sociodemographic information, physical health, and some chronic disease history of the subjects in the 2011–2014 cohort [25]. After screening according to the inclusion and exclusion criteria in Figure 1, a total of 2873 subjects were finally included in this study.

All subjects signed informed consent for baseline and follow-up surveys. The project was approved by the Biomedical Ethics Committee of Peking University, China (IRB00001052-13074).

### 2.2. Assessment of Dietary Pattern

To determine the main dietary patterns, based on the 13 food groups in CLHLS research, we used the principal component analysis method of exploratory factor analysis, according to Kaiser’ criterion, factors with eigenvalues greater >1 were extracted, and an orthogonal transformation was performed to rotate the factors (varimax rotation) to simplify the structure and to obtain greater interpretability. The naming of dietary patterns was based on the characteristics of the food groups in each factor combined with the interpretation of previous studies.

The factor scores for each pattern (dietary pattern score) were calculated by weighting the intake of food groups by their factor loadings. Higher dietary pattern scores indicated a higher preference for a specific dietary pattern. The subjects were divided into low-to high-tertile groups (Tertile 1, Tertile 2 and Tertile 3) based on their dietary pattern score in 2011.

### 2.3. Assessment of Depression

The PhenX (consensus measures for Phenotypes and eXposures) Toolkit was employed by CLHLS to assess depression (PhenX code: 120500). PhenX utilized the Composite International Diagnostic Interview Screening Scales (CIDI-SC), which was developed by the criteria of the Diagnostic and Statistical Manual of Mental Disorders (5th ed.; DSM-5). PhenX is a two-item, short self-report screening tool, which includes two levels of indicators, with a “yes” answer to any question indicating a representation of depression. This measurement has been applied to some previous studies [26,27]. The two questions were as follows:

Question 1: Have you had a time in last 12 months when you felt sad, blue, or depressed for two weeks or more? (Yes = 1; No = 0)

Question 2: Have you had a time in last 12 months lasting two weeks or more when you lost interest in most things like hobbies, work, or activities that usually give you pleasure? (Yes = 1; No = 0)

We used the Spearman–Brown coefficient instead of using Cronbach’s alpha to assess the reliability of the Phen X [28]. The Spearman–Brown coefficient was 0.712, indicating the good reliability and internal consistency of the tool for measuring depression.

### 2.4. Assessment of Covariates

According to the previous studies, we included sociodemographic information (gender, age, residence, living conditions, marital status, region residence, family income), physical health, living habits (body mass index (BMI), alcohol consumption, smoking status, exercise), and the number of chronic diseases (hypertension, diabetes, heart disease, stroke or cardiovascular disease (CVD), arthritis, rheumatism or rheumatoid disease, dyslipidemia, etc.) at baseline as covariates of this study.

### 2.5. Statistical Analyses

The initial fundamental characteristics of the groups, including gender, age, region of residence, marital status, family income, living conditions, BMI, alcohol consumption status, smoking status, exercise and the number of chronic diseases, were examined using independent chi-square testing.

The association between dietary patterns and risk of depression was summarized with odds ratios (*OR*) and their 95% confidence interval (*CI*) obtained from logistic regression. Two logistic models were built: the first one was adjusted for gender, age, marital status, region residence, family income and living conditions, and the second model was additionally adjusted for the rest of the variables described above. Adherence to the empirically derived dietary pattern was classified into tertiles, and the lowest tertile was used as the reference group. Because the conclusions of previous studies on male–female differences between dietary patterns and depression were inconsistent [29,30], subgroup analysis was performed by gender at baseline to test the differences in the association between dietary patterns and the risk of depression among men and women. Interactions were formally tested using the adjusted Wald test.

Database establishment, data cleaning, and statistical analysis were all completed by SPSS 26.0 and Stata 11.0. *p* < 0.05 was considered statistically significant.

## 3. Results

This study passed the Kaiser–Meyer–Olkin test, and the sampling adequacy value of the 13 food groups was 0.796 > 0.7. The result of the Bartlett sphere test showed that *χ*^2^ = 4839.439 (*p* < 0.001), indicating that the 13 food groups were not independent and were strongly correlated so that principal component analysis could be performed. Kaiser’ criterion finally identified three factors. The eigenvalues of the three factors after maximum rotation were 2.068, 1.708 and 1.627, respectively, and they explained 15.906%, 13.135% and 12.515% of the total variance; the cumulative contribution rate was 41.555% (Table A1 and Table A2).

According to the food characteristics of each of the three patterns, they were named the vegetable–egg–beans–milk pattern (fresh vegetable, eggs, bean products, and milk products), meat–fish pattern (meat and fish), and salt-preserved vegetable–garlic pattern (salt-preserved vegetables and garlic) (Table A3 and Table A4).

A total of 2873 subjects without depression symptoms at baseline were included in this study. Their mean age was (80.30 ± 0.18), 46.26% were female, 82.25% were rural residents, and the mean BMI was 22.05 kg/m^2^. For the vegetable–egg–beans–milk pattern, there were differences in the distribution of age, region of residence, family income, living conditions, BMI, smoking status, alcohol consumption status, exercise and the number of chronic diseases. For the meat–fish pattern, there were differences in the distribution of gender, age, marital status, region of residence, family income, living conditions, BMI, smoking status, alcohol consumption status and exercise. For the salt-preserved vegetable–garlic pattern, there were differences in the distribution of gender, age, marital status, region of residence, family income (except for Tertile 1), living conditions, BMI, smoking status, alcohol consumption status and exercise (Table 1).

During a mean follow-up of four years, we ascertained 346 subjects with depressive symptoms. Results from models with partial (model 1) and full (model 2) adjustments were rather similar. After controlling all the covariates, subjects in the second and third tertile of the vegetable–egg–beans–milk pattern score had a reduced risk of depression compared to subjects in the lowest tertile (OR = 0.61, *p* < 0.001) (OR = 0.65, *p* < 0.001). Subjects with the second tertile of the salt-preserved vegetable–garlic pattern score in 2014 had a higher risk of depression after 4 years than those in the lowest tertile (OR = 1.33, *p* < 0.001). No association was observed between meat-fish pattern and depression. (Table 2).

Subgroup analysis showed that the protective effect of the vegetable-egg-beans-milk pattern on depression was consistent in both men and women (*p* = 0.440). As shown in Table 3, the second tertile of the salt-preserved vegetable–garlic pattern score was significantly associated with depression in men (1.63 (1.06–2.49)), and the relationship between the third tertile of the pattern score and depression was borderline significant in women (1.34 (0.91–1.98)). The results of the men and women interaction term showed that the negative effect of the salt-preserved vegetable–garlic pattern on depression may not differ by gender either (*p* = 0.170).

## 4. Discussion

In this cohort study of old adults in China, we used exploratory factor analysis to identify three dietary patterns: the vegetable–egg–beans–milk pattern, meat–fish pattern, and salt-preserved vegetable–garlic pattern. Then, we examined the association between these three dietary patterns and the risk of depression in older adults. Our results showed that the vegetable–egg–beans–milk pattern score was associated with lower risk of depression after 4 years, the salt-preserved vegetable–garlic pattern was associated with higher risk of depression over time, and the meat–fish pattern was not observed to be related to the risk of depression in older adults. At the same time, there were no gender differences in these associations.

In this study, the vegetable–egg–beans–milk pattern was associated with reduced risk of depression. Our finding was similar to the other results. A cross-sectional study conducted in Australia found a link between high intake of a plant-based diet related to the Mediterranean diet and fewer depression symptoms [31], and a systematic review also showed that adherence to vegetables might reduce the development and progression of depression [32]. The protective effects of this pattern may include a variety of plant chemicals such as folic acid, dietary fiber, and antioxidant compounds, which can be used by participating in the synthesis and metabolism of neurotransmitters to relieve inflammation and stimulate the stress response system to regulate emotions [12,33,34]. It was also possible that these nutrients work together to produce a synergistic response to influence the likelihood of the depression arising [35]. At the same time, studies have proved that eggs included tryptophan, which may be converted to serotonin to regulate mood. At the same time, egg yolk is a good source of vitamin D and plays a vital role in reducing symptoms in elderly depressed patients [36,37,38]. Moreover, increasing the intake of milk and dairy products, especially low-fat milk intake, was also associated with a better mood [39]. Beans, which were also tryptophan-rich, have also been shown to reduce the risk of depression [40], and the trace element in the beans, especially zinc, was a nutrient widely associated with depression [41]. A study of Chinese adults found that moderate consumption of soy foods may reduce depressive symptoms, while relatively higher intakes may have the opposite effect [42]. However, the mechanism of the joint effect of different food on depression needs further clinical trials to examine.

We also observed no significant correlation between the risk of depression in the meat–fish pattern. Current evidence showed inconsistent results for the association between fish [43,44], omega-3 fatty acid intake [14,45,46] and depression. There are also controversial reports on meat intake and depressive symptoms [47,48]. The uncertain association between this pattern and psychological factors may be due to cultural differences in diet, lower meat consumption in the Chinese population, especially older people compared to Westerners, and the relatively narrower range of variation in animal food intake in our population than other reports [49,50]. Thus, the relationship between the meat–fish pattern and depression needs further exploration.

Salt-preserved vegetables and garlic are common on Chinese dining tables. Our analysis suggested that the salt-preserved vegetable–garlic pattern was associated with increased risk of depression. To our knowledge, there were no studies on the relationship between salt-preserved vegetables and depression. Even so, a cohort study reported a positive association between processed dietary patterns (such as preserved food) and the risk of depression in women [51]. It is well known that a large intake of salt-preserved vegetables may increase the risk of cardiovascular disease due to the high amount of salt. Patients with cardiovascular disease are more depressed than the general population [52], suggesting another way for the diet to regulate mental health. As for garlic, an experimental study demonstrated that garlic extract inhibited the monoamine oxidase levels, thus reducing the risk of depression due to the depletion of the monoamine neurotransmitters [53]. However, since our study explored the combined pattern in which these two food categories were the dominant characteristics, it was unclear whether garlic played a positive role in this dietary pattern and should be further explored in the future.

Previous studies of the relationship between dietary patterns and depression have yielded inconsistent results regarding differences between men and women. A Japanese cross-sectional study reported that the plant-based food–fish product pattern and meat pattern were significantly associated with depression in men, but no association was observed in women [29]. A cross-sectional study in the US reported that the dietary pattern of vegetables, fruits, fish, and whole grains was significantly associated with depression scores in women after adjusting for covariates. In contrast, this association was not observed in men [30]. Even so, the dietary patterns they explored may be suitable for only part of the population, our study found that the vegetable–egg–beans–milk pattern was associated with a lower risk of depression in older adults, and this association had no gender difference. Moreover, the salt-preserved vegetable–garlic pattern was associated with a higher risk of depression in men and it may be also associated with increased depression risk in women, and this negative effect may be greater in men. More studies should be conducted to further examine the gender differences in this pattern.

More randomized controlled trials are needed to further prove the validity of the dietary patterns explored in observational studies. Previous clinical trials have confirmed the effectiveness of the Mediterranean dietary pattern and nutritional supplements in improving depression [54,55], but some of the dietary patterns defined by later studies also need to be validated. Moreover, how to make older adults adhere to a beneficial diet against depression is also a point worth exploring. In a trial study, an intervention based on individual telephone calls and personalized dietary advice increased adherence to the Mediterranean dietary pattern among patients with coronary artery disease [56]. However, there are few studies on interventions to increase dietary pattern adherence in the elderly, and this research field needs to be expanded in the future.

The strength of this study was that it was a 4-year cohort study rather than focusing only on cross-sectional dietary intake, which allowed us to investigate the impact of long-term dietary patterns on the risk of sensitivity to depression. Second, the CLHLS, which covers 23 provinces or municipalities, provided the data. These regions varied in geography, economic development, public resources and health indicators and were more representative of Chinese residents.

Several limitations also existed in this study. First, all the answers to the questionnaire were self-reported by the subjects, which were prone to recall bias. Second, the questionnaire included only the frequency of food intake without the amount of food intake. The frequency may not completely match the total intake, but intake frequency may be more important than the intake amount [57]. Third, this study was conducted on older Chinese adults, and the results cannot be extrapolated to other races. Finally, although we have adjusted for many potential confounders, due to the common risk factors, this observational study cannot exclude the existence of possible reverse causality and the influence of residual confounding.

## 5. Conclusions

In conclusion, our longitudinal study revealed that the vegetable–egg–beans–milk dietary pattern was associated with a lower risk of depression among Chinese older adults, as well as the salt-preserved vegetable–garlic dietary pattern was associated with a higher risk of depression. The meat–fish pattern was not found to associate with depression in the elderly. Moreover, there were no gender differences in these findings. This study provided evidence that a diet was conducive to preventing depression in the elderly.

## Figures and Tables

**Table 1:** Characteristics of the non-depression population at baseline by adherence to the dietary pattern.

Characteristic	Vegetable–Egg–Beans–Milk Pattern			Meat–Fish Pattern			Salt-Preserved Vegetable–Garlic Pattern		
	Tertile1	Tertile2	Tertile3	Tertile1	Tertile2	Tertile3	Tertile1	Tertile2	Tertile3
Gender, %									
Male	54.10	52.50	54.50	**47.10**	**54.30**	**59.80**	**47.70**	**55.50**	**58.00**
Female	45.90	47.50	45.50	**52.90**	**45.70**	**40.20**	**52.30**	**44.50**	**42.00**
Age, %									
≥60 and <75	**32.80**	**38.10**	**31.30**	**31.30**	**30.3v**	**40.60**	**27.00**	**35.70**	**39.50**
≥75 to <85	**35.70**	**34.30**	**32.70**	**33.50**	**37.80**	**31.30**	**32.70**	**34.40**	**35.50**
≥85	**31.50**	**27.60**	**36.00**	**35.10**	**31.90**	**28.10**	**40.30**	**29.90**	**25.00**
Marital status, %									
Married or partnered	50.90	54.50	54.10	**46.60**	**52.10**	**61.20**	**44.80**	**55.30**	**59.60**
Unmarried or others	49.10	45.50	45.90	**53.40**	**47.10**	**38.80**	**55.20**	**44.70**	**40.40**
Region of residence, %									
Urban community	**7.20**	**14.40**	**31.00**	**12.50**	**17.90**	**22.90**	**15.10**	**17.60**	**20.50**
Rural village	**92.80**	**85.60**	**69.00**	**87.50**	**82.10**	**77.10**	**84.90**	**82.40**	**79.50**
Family income, %									
Quintile 1 (lowest)	**25.10**	**20.40**	**15.10**	**31.20**	**16.50**	**12.70**	23.10	22.10	15.40
Quintile 2	**24.20**	**19.80**	**14.80**	**21.20**	**21.00**	**16.40**	**20.20**	**19.20**	**19.30**
Quintile 3	**18.00**	**21.20**	**20.20**	**17.10**	**21.40**	**20.90**	**17.90**	**19.80**	**21.80**
Quintile 4	**14.70**	**20.00**	**26.20**	**15.70**	**21.40**	**24.00**	**20.20**	**19.40**	**21.40**
Quintile5 (highest)	**17.90**	**18.60**	**23.70**	**14.80**	**19.70**	**26.00**	**18.70**	**19.50**	**22.10**
Living conditions, %									
Alone	**77.10**	**83.90**	**81.50**	**75.80**	**81.00**	**85.80**	**76.10**	**80.70**	**85.80**
Not alone	**22.90**	**16.10**	**18.50**	**24.20**	**19.00**	**14.20**	**23.90**	**19.30**	**14.20**
BMI, %									
Underweight	**19.80**	**17.80**	**13.80**	**17.80**	**17.90**	**15.40**	**21.60**	**16.60**	**13.10**
Normal	**57.60**	**55.60**	**54.60**	**58.70**	**55.70**	**53.40**	**54.80**	**55.40**	**57.50**
Overweight	**17.50**	**20.10**	**23.00**	**17.40**	**19.50**	**23.80**	**17.50**	**21.30**	**21.90**
Obese	**5.10**	**6.50**	**8.60**	**6.10**	**6.90**	**7.40**	**6.10**	**6.70**	**7.50**
Smoking status, %									
Current	**26.80**	**23.50**	**18.90**	**20.30**	**22.30**	**26.60**	**16.20**	**25.30**	**27.50**
Former	**13.40**	**12.60**	**19.70**	**14.10**	**14.20**	**17.60**	**13.30**	**14.60**	**17.80**
Never	**59.80**	**63.90**	**61.40**	**65.60**	**63.50**	**55.80**	**70.50**	**60.10**	**54.60**
Alcohol consumption status, %									
Current	**21.70**	**21.60**	**18.1**	**15.9**	**20.70**	**24.80**	**12.70**	**20.60**	**27.90**
Former	**12.40**	**11.30**	**17.0**	**11.8**	**13.30**	**15.70**	**10.50**	**14.40**	**15.80**
Never	**65.90**	**67.10**	**64.9**	**72.3**	**66.00**	**59.50**	**76.80**	**65.00**	**56.30**
Exercise, %									
Yes	**38.20**	**37.30**	**55.3**	**37.5**	**43.40**	**50.40**	**39.40**	**44.40**	**47.30**
No	**61.80**	**62.70**	**44.7**	**62.5**	**56.60**	**49.60**	**60.60**	**55.60**	**52.70**
No. of chronic diseases, %									
0	**54.60**	**53.70**	**45.7**	48.6	53.00	52.20	52.60	51.60	49.60
1	**32.30**	**28.30**	**34.1**	33.6	30.90	30.20	31.30	30.00	33.30
≥2	**13.10**	**18.00**	**20.2**	17.8	16.10	17.60	16.10	18.40	17.10

Note: Bold font represented different statistical distribution between characteristics and dietary patterns.

**Table 2:** Association between dietary patterns and risk of depression during a 4-year follow-up of older adults.

Model	Vegetable–Egg–Beans–Milk Pattern			Meat–Fish Pattern			Salt-Preserved Vegetable–Garlic Pattern		
	Tertile1	Tertile2	Tertile3	Tertile1	Tertile2	Tertile3	Tertile1	Tertile2	Tertile3
	OR (95%CI)	OR (95%CI)	OR (95%CI)	OR (95%CI)	OR (95%CI)	OR (95%CI)	OR (95%CI)	OR (95%CI)	OR (95%CI)
Crude model	Ref.	0.65 (0.49–0.86) *	0.78 (0.60–1.02)	Ref.	0.81 (0.61–1.07)	0.97 (0.74–1.28)	Ref.	1.31 (0.99–1.73)	1.25 (0.95–1.66)
Adjusted model 1	Ref.	0.61 (0.46–0.81) *	0.66 (0.50–0.88) *	Ref.	0.80 (0.60–1.07)	0.95 (0.72–1.26)	Ref.	1.32 (0.99–1.76)	1.25 (0.95–1.68)
Adjusted model 2	Ref.	0.61 (0.46–0.82) *	0.65 (0.49–0.87) *	Ref.	0.82 (0.62–1.09)	0.97 (0.73–1.29)	Ref.	1.33 (1.00–1.77) *	1.27 (0.95–1.70)

Note: *: *p* < 0.05. Crude model: no adjustment; Model 1, adjusted for gender, age, marital status, region residence, family income and living conditions; Model 2, Model 1 + BMI, smoking status, alcohol consumption status, exercise and the number of chronic diseases.

**Table 3:** Differences in the association between dietary patterns and the risk of depression among men and women.

**Group**	**Vegetable–Egg–Beans–Milk Pattern**			***p* Value for** **Interaction**
	**Tertile 1**	**Tertile 2**	**Tertile 3**	
Gender				0.440
Male	Ref.	0.71 (0.47–1.06)	0.63 (0.41–0.97) *	
Female	Ref.	0.53 (0.35–0.80) *	0.63 (0.43–0.97) *	
	**Meat–Fish Pattern**			***p* Value for** **Interaction**
	**Tertile 1**	**Tertile 2**	**Tertile 3**	
Gender				0.870
Male	Ref.	0.95 (0.62–1.45)	1.14 (0.75–1.73)	
Female	Ref.	0.71 (0.48–1.06)	0.85 (0.57–1.26)	
	**Salt-Preserved** **Vegetable–Garlic Pattern**			***p* Value for** **Interaction**
	**Tertile 1**	**Tertile 2**	**Tertile 3**	
Gender				0.170
Male	Ref.	1.63 (1.06–2.49) *	1.29 (0.82–2.01)	
Female	Ref.	1.08 (0.72–1.61)	1.34 (0.91–1.98)	

Note: *: *p* < 0.05. All models adjusted for age, marital status, region residence, family income, living conditions, BMI, smoking status, alcohol consumption status, exercise and the number of chronic diseases.

## Data Availability

The raw data supporting the conclusions of this article can be found here: https://opendata.pku.edu.cn/dataverse/CHADS (accessed on 4 October 2022).

## Appendix A

**Table A1:** Kaiser–Meyer–Olkin Measure of Sampling Adequacy.

Kaiser–Meyer–Olkin Measure of Sampling Adequacy		0.796
Bartlett’s Test of Sphericity	Approx. chi-Square	4839.439
	df	79
	Sig.	<0.001

**Table A2:** Total Variance Explained.

Component	Rotation Sums of Squared Loadings		
	Total	% of Variance	Cumulative %
1	2.068	15.906	15.906
2	1.708	13.135	29.041
3	1.627	12.515	41.555

**Table A3:** The rotated composition matrix.

Food Groups	Component		
	Vegetable–Egg–Beans–Milk Pattern	Meat-Fish Pattern	Salt-Preserved Vegetable–Garlic Pattern
Fresh vegetable	0.505	-	-
Fresh fruit	-	-	-
Meat	-	0.741	-
Fish	-	0.699	-
Eggs	0.684	-	-
Bean products	0.524	-	-
Salt-preserved vegetable	-	-	0.744
Sugar	-	-	-
Tea	-	-	-
Garlic	-	-	0.616
Milk products	0.739	-	-
Nut products	-	-	-
Mushroom or algae	-	-	-

Extraction Method: principal component analysis. Rotation Method: Varimax with Kaiser Normalization. Coefficient display format: Small coefficients with absolute value <0.5 are not displayed.

**Table A4:** Factor load matrix of dietary pattern.

Food Groups	Component		
	Vegetable–Egg–Beans–Milk Pattern	Meat–Fish Pattern	Salt-Preserved Vegetable–Garlic Pattern
Fresh vegetable	0.234	0.185	−0.150
Fresh fruit	−0.113	0.309	0.061
Meat	−0.114	0.309	0.061
Fish	0.000	0.045	−0.138
Eggs	0.395	−0.113	−0.083
Bean products	0.257	−0.068	0.055
Salt-preserved vegetable	−0.220	−0.028	0.558
Sugar	0.073	−0.156	0.266
Tea	−0.106	0.192	0.209
Garlic	−0.031	−0.037	0.402
Milk products	0.448	−0.138	−0.140
Nut products	0.095	0.042	0.207
Mushroom or algae	0.181	0.052	0.133

Extraction Method: principal component analysis. Rotation Method: Varimax with Kaiser Normalization.
